# Severe Bioprosthetic Mitral Valve Stenosis and Heart Failure in a Young Woman with Systemic Lupus Erythematosus

**DOI:** 10.1155/2016/3250845

**Published:** 2016-08-17

**Authors:** Siddharth Wartak, Isaac Akkad, Adnan Sadiq, Gregory Crooke, Manfred Moskovits, Robert Frankel, Gerald Hollander, Jacob Shani

**Affiliations:** ^1^Department of Cardiology, Maimonides Medical Center, Brooklyn, NY 11219, USA; ^2^Department of Medicine, Maimonides Medical Center, Brooklyn, NY 11219, USA; ^3^Department of Interventional Cardiology, Maimonides Medical Center, Brooklyn, NY 11219, USA

## Abstract

A 23-year-old African American woman with a past medical history of systemic lupus erythematous (SLE), secondary hypertension, and end stage renal disease (ESRD) on hemodialysis for eight years was stable until she developed symptomatic severe mitral regurgitation with preserved ejection fraction. She underwent a bioprosthetic mitral valve replacement (MVR) at outside hospital. However, within a year of her surgery, she presented to our hospital with NYHA class IV symptoms. She was treated for heart failure but in view of her persistent symptoms and low EF was considered for heart and kidney transplant. This was a challenge in view of her history of lupus. We presumed that her stenosis of bioprosthetic valve was secondary to lupus and renal disease. We hypothesized that her low ejection fraction was secondary to mitral stenosis and potentially reversible. We performed a dobutamine stress echocardiogram, which revealed an improved ejection fraction to more than 50% and confirmed preserved inotropic contractile reserve of her myocardium. Based on this finding, she underwent a metallic mitral valve and tricuspid valve replacement. Following surgery, her symptoms completely resolved. This case highlights the pathophysiology of lupus causing stenosis of prosthetic valves and low ejection cardiomyopathy.

## 1. Introduction

Systemic Lupus Erythematosus (SLE) is an autoimmune disorder resulting in multisystemic inflammatory damage mediated by the production of autoantibodies and immune complexes against a variety of self-antigens.

The routine use of Doppler echocardiography in the evaluation of SLE mediated heart damage has disclosed a higher prevalence of patients with cardiac abnormalities than previously thought, given the high prevalence of clinically silent disease.

The use of dobutamine stress echocardiogram in the setting of nonischemic cardiomyopathy can be a very helpful tool to assess the inotropic contractile reserve of myocardium and predict superior outcomes.

## 2. Case

**Figure 1:**
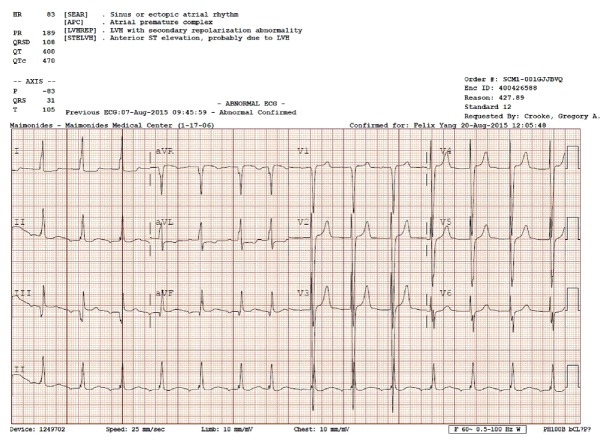
Electrocardiogram showed ectopic atrial rhythm, atrial premature complexes, left ventricular hypertrophy (LVH) with secondary repolarization abnormality, and anterior ST elevation, probably due to LVH.

A 23-year-old African American woman with a past medical history of systemic lupus erythematous, secondary hypertension, and end stage renal disease (ESRD) on hemodialysis for eight years was doing well. She developed progressive shortness of breath and was diagnosed with severe mitral regurgitation with preserved ejection fraction. She underwent successful bioprosthetic mitral valve replacement. About 6 months following surgery, she started feeling exertional shortness of breath and a year later she presented to our emergency department with NYHA class IV symptoms. Patient's heart rate was 86 bpm, blood pressure was 153/101, and respiratory rate was of 24 breaths per minute and she was afebrile. On auscultation, a holosystolic murmur and loud P2 were appreciated at the left lower sternal border. On ECG, there was left ventricular hypertrophy (LVH) with secondary repolarization abnormality (Figure 1).

The patient's hemoglobin was 5.8 gm/dL and her hematocrit was 17.9%, serum BNP was >4700 pg/mL, and negative cardiac biomarkers, positive dsDNA antibody, positive ANA, and low C3 and C4 levels were also present.

On echocardiogram, the bioprosthetic mitral valve was found with thickened calcified leaflets, restricted mobility, and severe prosthetic stenosis by Doppler, with a mean gradient of 21 mmHg and a prosthetic valve area of 0.45 cm^2^. The left ventricular systolic function was severely decreased with global cardiomyopathy and ejection fraction <20%. Severe tricuspid valve regurgitation was also seen. Patient was treated with hemodialysis, furosemide, metoprolol, nifedipine, and transfused 2 units of PRBCs, and started on prednisone and hydroxychloroquine. She was treated for heart failure but in view of her persistent symptoms and extremely low EF was considered for heart and kidney transplant.

Cardiac MRI showed global hypokinesis, IVC, and hepatic vein dilation consistent with tricuspid regurgitation, severe mitral stenosis, marked atrial dilatation of 6.5 cm, and marked ventricular dilatation of 7.7 cm. Cardiac catheterization showed normal coronaries and LVEDP was 5 mmHg, with LVEF of 25% with global hypokinesis and no LV-Ao gradient. On right heart catheterization, a mean RA pressure of 9 mmHg, RV of 34 mmHg, and PA of 49/27 mmHg and mean of 36 mmHg, with a wedge pressure of 27 with V 28 mmHg, were found. The patient had a mean gradient across her mitral valve of 17.24 mmHg and her mitral valve area was 0.63 cm^2^ estimated by Hakki formula. A dobutamine stress echocardiogram was performed to evaluate the contractile reserve of the left ventricle and showed dramatically improved ejection fraction to more than 50%. Because of these findings on dobutamine stress echocardiogram, patient was considered for redoing valve surgery rather than pursuing for transplant. She underwent successful mechanical mitral and tricuspid valve replacement. Postoperative period and discharge were uneventful. Her symptoms improved and she returned to college to proceed with the routine activities. A follow-up echocardiogram after a month showed normal LV cavity size with ejection fraction of 60% and normal functioning prosthetic valves.

## 3. Discussion

We herein describe a young patient with systemic lupus erythematous (SLE) and ESRD developing severe MR. SLE is an autoimmune disorder resulting in multisystemic inflammatory damage mediated by the production of autoantibodies and immune complexes against a variety of self-antigens [1, 2]. There is a high prevalence of up to 50% of cardiac involvement in patients with SLE [3]. The most characteristic cardiac abnormalities seen in SLE are noninfective vegetations (Libman-Sacks endocarditis) present in up to 50% of patients at necropsy and pericarditis present in up to 25% of patients [4–7]. Valve dysfunctions, such as aortic stenosis, aortic insufficiency, mitral stenosis, and mitral insufficiency, occasionally manifest during life and rarely may necessitate surgery [8–10].

The routine use of Doppler echocardiography in the evaluation of SLE mediated heart damage has disclosed a higher prevalence of patients with cardiac abnormalities than previously thought, given the high prevalence of clinically silent disease [7–11].

Among valvular heart disease, mitral regurgitation is the most common manifestation [2, 4, 5, 10]. Our patient underwent a successful valve replacement but unfortunately developed accelerated degeneration of bioprosthetic valve within a year. The stenosis within one year was likely secondary to combination of lupus and renal disease [6, 8, 12, 13]. The ideal selection of bioprosthetic or metallic valve is not known as both valves have there own associated morbidity and mortality [14–17]. In our patient, her first surgery was with bioprosthetic valve as her personal preference over a metallic valve. She was planning for future pregnancy and did not want to take anticoagulation. However, we used mechanical valves for her second surgery as she had undoubtedly failed bioprosthetic valves. The explanation of low ejection fraction was challenging. It is well recognized that there is increased afterload following mitral valve replacement for mitral regurgitation and it is not uncommon to see decreased ejection fraction following replacement surgery [6, 10, 12, 13].

However, this drop in EF is very marginal unlike the case in our patient. In our patient, lupus was in control making lupus myocarditis low on the differential. We hypothesize that the low EF was secondary to mitral stenosis. This is from altered loading condition with decreased preload and increased vasoconstriction from increased afterload.

Lastly, the use of dobutamine stress echocardiography in cardiomyopathy has shown prognostic implications in nonischemic cardiomyopathy [10, 18]. We wanted to maximize the utility of this prediction in our patient and indeed the results showed improved EF to more than 50%.

This case illustrates and discusses three key aspects. First, it emphasizes the influence of lupus on heart causing predominantly valvular disorders: mitral regurgitation of native valve and stenosis of bioprosthetic valves. Secondly, it highlighted the pathophysiology of mitral stenosis: native or prosthetic, both causing a decreased preload and increased afterload situation causing a low ejection fraction. Finally, the use of dobutamine stress echo was a very helpful tool to assess the inotropic contractile reserve of cardiomyopathy and predict superior outcome. In our patient, the redo of valve surgery corrected the pathophysiology of stenosis and strikingly improved her myocardial function. She did extremely well clinically and we could elude an aggressive, unnecessary, and probably impractical heart transplant surgery in this young woman with heart failure.
